# Gamma knife radiosurgery for renal cell carcinoma brain metastases across systemic therapy eras: survival, intracranial failure, and lesion-level predictors

**DOI:** 10.1007/s11060-026-05692-3

**Published:** 2026-07-03

**Authors:** Neil D. Almeida, Sarthak Sinha, Mengyu Fang, Harshini Cheruvu, Roshan Tasgaonkar, Babar Gulzar, Venkatesh Madhugiri, Victor Goulenko, Dheerendra Prasad

**Affiliations:** 1 Department of Radiation Medicine, Roswell Park Comprehensive Cancer Center, 665 Elm St, Buffalo, NY 14203 USA; 2 Department of Statistics & Data Science, Cornell University, Ithaca, NY USA; 3 Department of Biostatistics & Bioinformatics, Roswell Park Comprehensive Cancer Center, Buffalo, NY USA; 4 Department of Neurosurgery, Roswell Park Comprehensive Cancer Center, Buffalo, NY USA; 5 Department of Radiation Oncology, Jacobs School of Medicine and Biomedical Sciences, University at Buffalo, Buffalo, NY USA

**Keywords:** Renal cell carcinoma, Brain metastases, Gamma knife radiosurgery, Tyrosine kinase inhibitors, Immunotherapy, Postoperative cavity, Local control

## Abstract

**Background:**

Brain metastases from renal cell carcinoma (RCC) are uncommon but clinically consequential. We evaluated survival, intracranial failure, lesion-level local failure, and MRI volumetric response after Gamma Knife radiosurgery (GKRS), including associations with systemic therapy exposure.

**Methods:**

We retrospectively identified patients treated with GKRS for intracranial RCC metastases (2001–2025). Systemic therapy exposure (TKI and/or immunotherapy) was captured time-agnostically. Overall survival (OS) and patient-level intracranial treatment failure were analyzed using Kaplan–Meier/Cox models. Lesion-level treatment failure was analyzed using clustered generalized estimating equations (GEE). Six-month volumetric response was assessed using an epsilon-stabilized log volume ratio ln{(V_6m_+ϵ)/(V_GKRS_+ϵ)}, ε = 0.01.

**Results:**

Thirty-four patients (87 lesions) were treated; 22 deaths occurred. Overall survival was numerically longer among patients who received systemic therapy compared with those who did not, although this did not reach statistical significance (log-rank *p* = 0.058). In a parsimonious adjusted Cox model, TKI exposure was associated with improved OS (aHR 0.191; 95% CI 0.062–0.593; *p* = 0.004), as was higher KPS (HR 0.37 per 10-point increase; 95% CI 0.21–0.63; *p* < 0.001). Ten patient-level intracranial treatment failure events occurred, without significant differences by systemic therapy exposure. At the lesion level, 15/87 lesions failed; postoperative cavities had higher failure than intact lesions (71.4% vs. 12.5%; *p* = 0.001) and remained associated with failure after clustered adjustment (OR 9.92; 95% CI 1.44–68.27; *p* = 0.020). Dmax was not correlated with 6-month log volume ratio (ρ=−0.075; *p* = 0.535), but poorer 6-month volumetric response was associated with subsequent lesion failure (*p* = 0.0023; clustered OR 2.84; 95% CI 1.16–6.96; *p* = 0.023).

**Conclusions:**

In RCC brain metastases treated with GKRS, postoperative cavities showed a higher observed rate of local failure than intact lesions, although this finding should be interpreted cautiously given the small number of cavity targets. While Dmax was not associated with early volumetric change, early 6-month volumetric trajectory was associated with subsequent local failure, suggesting a pragmatic imaging marker for risk-adapted surveillance and salvage planning. Systemic therapy exposure, particularly TKIs, was associated with OS in adjusted analysis, whereas intracranial treatment failure did not differ by systemic exposure in this cohort.

**Supplementary Information:**

The online version contains supplementary material available at 10.1007/s11060-026-05692-3.

## Introduction

Renal cell carcinoma (RCC) accounts for approximately 90% of kidney malignancies and continues to rise in incidence worldwide [1–6]. A substantial proportion of patients develop metastatic disease, either at presentation or after treatment for localized RCC, with the lungs, bone, liver, and lymph nodes representing the most common sites [3–6]. Brain metastases are less frequent, reported in roughly 4–11% of RCC cases, but confer disproportionately poor outcomes and represent a clinically distinct subset with unique management challenges [2, 5, 7]. 

Local therapy remains central to the management of RCC brain metastases. Surgical resection is commonly used for symptomatic lesions, hemorrhagic metastases, or tumors with significant mass effect, while stereotactic radiosurgery (SRS) has become a preferred modality for many patients because of its ability to deliver highly conformal radiation with sparing of normal brain [8, 9]. Outcomes after SRS for RCC brain metastases have been favorable in multiple series; however, survival remains limited for many patients, reflecting the dominant influence of extracranial disease course and patient fitness [10]. In parallel, postoperative cavity targets represent a distinct radiosurgical substrate, often larger, irregular, and dynamically changing, where local control can be more challenging than for intact metastases [11–13]. 

Systemic therapy for RCC has evolved substantially over the last two decades, with tyrosine kinase inhibitors (TKIs) and immune checkpoint inhibitors (ICIs) becoming foundational components of metastatic RCC management [14]. Growing clinical experience suggests that integrating systemic therapy with SRS may improve outcomes in select populations, and retrospective series have reported improved overall survival among patients receiving targeted agents in conjunction with radiosurgery, while also raising concerns regarding toxicity such as radiation necrosis in some contexts [15–17]. More recently, immunotherapy-based strategies combined with SRS have shown encouraging results, including analyses using propensity-based methods in RCC brain metastases [18]. Despite this progress, optimal integration of systemic therapy with radiosurgery remains incompletely defined, particularly in real-world cohorts treated across systemic therapy eras and with heterogeneous intracranial targets [14, 17, 19, 20]. 

In this context, we conducted a retrospective cohort study of patients with intracranial RCC metastases treated with Gamma Knife radiosurgery (GKRS) to evaluate patient-level overall survival and intracranial treatment failure, as well as lesion-level local failure with particular attention to postoperative cavity status. We additionally analyzed serial MRI-based volumetrics to characterize early response patterns and explore whether early volumetric trajectories relate to subsequent local failure in this radioresistant histologic subtype.

## Methods

### Study design and patient cohort

We performed a retrospective cohort study of patients treated with GKRS for intracranial metastases from RCC. The institutional registry was queried for treatments delivered between January 1, 2001 and February 28, 2025. This retrospective study was conducted in accordance with the Declaration of Helsinki, as revised in 2013, and all methods were carried out in accordance with relevant guidelines and regulations. The study protocol was approved by the Roswell Park Comprehensive Cancer Center Institutional Review Board (EDR-103707).

A total of 34 patients with 87 intracranial lesions treated with GKRS were identified. Surgical resection was performed in 7 patients (7 lesions). GKRS was delivered as upfront definitive therapy in 27 patients and as adjuvant therapy after resection in 7 patients.

### Radiosurgery technique

GKRS was performed using Leksell Gamma Knife models type C, Perfexion, ICON, or Esprit (Elekta Instrument AB, Stockholm, Sweden). Prescription dose and plan parameters were individualized based on lesion size/volume and clinical considerations.

Prescription dose was selected by the treating radiation oncologist and neurosurgeon based on target volume, location, prior surgery, proximity to organs at risk, and expected tolerance of adjacent brain. For intact metastases, single-fraction GKRS was generally prescribed with dose reduction for larger lesions or lesions adjacent to critical structures. Postoperative cavity plans were individualized according to cavity size, and shape. However, there was no uniform institutional policy mandating dose escalation solely for RCC histology. The higher observed Dmax among cavity targets likely reflects larger and more irregular target geometry and treatment planning heterogeneity across the 24-year study period rather than a standardized cavity dose-escalation protocol.

The 87 treated lesions represent all intracranial RCC metastases treated with GKRS during the study period. Patients could contribute more than one lesion, and lesions could be treated either during a single GKRS session or across separate GKRS courses when new or progressive lesions were identified. Overall, 9 patients underwent one GKRS course and 25 patients underwent more than one GKRS course. All patient-level time-to-event analyses were anchored to the date of first GKRS, whereas lesion-level analyses were performed at the treated-lesion level with clustering by patient ID.

### Systemic therapy exposure definitions

Systemic therapy was categorized by class as tyrosine kinase inhibitors (TKI) and/or Immunotherapy (IO). Because timing relative to GKRS was not reliably captured, systemic therapy was treated as a time-agnostic exposure and categorized as TKI only, IO only, TKI plus IO, or no systemic therapy. For analyses, systemic therapy was additionally evaluated as (1) any systemic therapy (TKI and/or IO) vs. none, and (2) TKI exposure vs. no TKI exposure; these systemic therapy comparisons were prespecified as exploratory due to potential confounding by treatment selection and sequencing.

Disease burden was categorized as high versus low based on extracranial disease status. High disease burden was defined as the presence of widespread and/or progressive extracranial metastatic disease at the time of GKRS; low disease burden was defined as absence of widespread/progressive extracranial disease.

### Follow-up and imaging assessment

Patients underwent clinical and MRI follow-up at institutional intervals (approximately 6 months, 1 year, and 2 years) and at the last known follow-up. Lesion volumes were recorded on serial MRI to assess post-treatment volumetric response.

Follow-up timing reflected real-world institutional practice and varied across the long study period. Although many contemporary SRS protocols obtain MRI every 2–4 months after treatment [21, 22], this retrospective registry captured volumetric follow-up at standardized landmark intervals of approximately 6 months, 1 year, 2 years, and last follow-up. Therefore, the 6-month volumetric analysis was intended to evaluate an early landmark imaging trajectory rather than the exact time of failure onset.

### Outcomes

The primary endpoint was overall survival (OS), defined at the patient level as time from the first GKRS to death from any cause. A key secondary patient-level endpoint was intracranial treatment failure, defined as time from first GKRS to intracranial failure per the registry variable.

Secondary lesion-level endpoints included treatment failure (local failure) and volumetric response. Lesion-level treatment failure was defined as radiographic progression of a treated lesion and/or the need for salvage re-irradiation with GKRS for that lesion. Progression was assessed using radiographic-only RANO-BM principles where feasible [23]. (e.g., progression defined by ≥ 20% increase in sum of longest diameters relative to nadir with an absolute increase of ≥ 5 mm and/or new intracranial lesions). The requirement for an absolute increase of ≥ 5 mm was an author-defined modification intended to reduce misclassification from measurement variability in very small treated lesions, which were common in this cohort. Because volumetric thresholds were not prespecified, volumetric response analyses were treated as exploratory. Exploratory dosimetric analysis evaluated the association between Dmax and lesion volumetric change on follow-up imaging.

### Statistical analysis

Overall survival was estimated using Kaplan–Meier methods and compared using log-rank tests. Cox proportional hazards regression was used for OS modeling. Given the sample size and event counts, multivariable modeling was parsimonious, with the primary adjusted model including TKI exposure, age, KPS, and disease burden; univariate Cox models were also fit for systemic therapy variables.

Patient-level intracranial treatment failure was analyzed using Kaplan–Meier methods/log-rank tests and Cox regression (univariate and the same parsimonious adjusted model). TKI exposure was selected for the primary parsimonious adjusted OS model because targeted therapy represented the earliest systemic therapy class consistently captured across the study period and because prior RCC brain metastasis radiosurgery series have reported survival associations with targeted agents [16, 24]. Any systemic therapy and immunotherapy exposure were evaluated in univariable analyses and interpreted as exploratory because of overlap between TKI and immunotherapy exposure, limited events, and risk of model overfitting.

Lesion-level treatment failure was analyzed as a binary endpoint. Because lesion-level time-to-failure was not consistently available, inferential modeling used generalized estimating equations (GEE) logistic regression with robust sandwich standard errors clustered by patient ID (exchangeable working correlation). Prespecified predictors included cavity status, log-transformed baseline lesion volume, and Dmax. For descriptive comparisons of cavity vs. intact lesions, Fisher’s exact test was used for categorical outcomes, and Mann–Whitney U tests were used for continuous variables (baseline volume, Dmax). For volumetric response, the primary analysis used early volumetric trajectory, defined as the epsilon-stabilized log volume ratio at approximately 6 months:

$$1\mathrm{n}\,\{{(\mathrm{V}_{6\mathrm{m}}+\varepsilon)/(\mathrm{V}_\mathrm{GKRS}+\varepsilon)}\},$$

where V_6m_ represents lesion volume at approximately 6-month follow-up, V_GKRS_ represents baseline lesion volume at GKRS, and ε = 0.01 was applied to reduce instability near zero. More negative values indicate greater early volumetric regression, whereas higher values indicate less regression or interval growth.

Association between Dmax and volumetric response was assessed using Spearman’s rank correlation. To reduce pseudoreplication from multiple lesions per patient, a prespecified sensitivity analysis was performed using an index lesion per patient (largest baseline treated lesion among those with 6-month follow-up). The primary volumetric analyses used all eligible lesions with 6-month follow-up. Because multiple lesions within a patient are not statistically independent, we also performed a sensitivity analysis restricted to one index lesion per patient, defined as the largest baseline treated lesion among lesions with 6-month follow-up. This sensitivity analysis was not intended to replace the full lesion-level analysis, but to assess whether the direction of findings persisted after reducing within-patient pseudoreplication. All tests were two-sided, and statistical analyses were performed in R (version 4.3.2) and SPSS 28.0.1.0.

## Results

### Cohort characteristics

Between January 1, 2001 and February 28, 2025, 34 patients underwent GKRS for 87 intracranial RCC metastases. Systemic therapy exposure (time-agnostic, per available registry capture) included TKI and/or immunotherapy, with patients categorized as TKI only, immunotherapy only, TKI plus immunotherapy, or no systemic therapy (Table 1). The most frequently recorded agents were nivolumab and cabozantinib (Supplementary Table 1).

**Table 1:** Patient and treatment characteristics

Characteristic	Overall (*N* = 34)	No systemic (*N* = 11)	Any systemic (*N* = 23)	*p*-value
Age, years (median [IQR])	60.5 (14)	63.0 (19)	57.0 (13)	0.29
Male sex, n (%)	23 (67.6%)	7 (63.6%)	16 (69.6%)	1.0
KPS (median)	80.0	80.0	80.0	0.67
ECOG (median)	1.0	1.0	1.0	0.38
Surgery, n (%) - No Sx	27 (79.4%)	11 (100%)	16 (69.6%)	0.11
Surgery, n (%) - Yes Sx	7 (20.6%)	0 (0.0%)	7 (30.4%)	0.06
High disease burden, n (%)	27 (79.4%)	10 (90.9%)	17 (73.9%)	0.38
Smoking history, n (%)	17 (50.0%)	6 (54.5%)	11 (47.8%)	1.0
No. lesions per patient (median)	1.5	2.0	1.0	0.02
Systemic therapy: No systemic, n (%)	11 (32.4%)			
Systemic therapy: TKI only, n (%)	2 (5.9%)			
Systemic therapy: IO only, n (%)	5 (14.7%)			
Systemic therapy: TKI + IO, n (%)	16 (47.1%)			

Baseline characteristics of patients undergoing GKRS for RCC brain metastases, overall and stratified by receipt of any systemic therapy (Tyrosine Kinase Inhibitor (TKI) and/or immunotherapy(IO)) versus none. Continuous variables are reported as median (IQR) and compared using Wilcoxon rank-sum tests; categorical variables are reported as n (%) and compared using Fisher’s exact tests. High disease burden was defined as widespread extracranial metastatic disease at the time of GKRS

In an exploratory era-stratified analysis, 8 patients were treated in the earlier era/pre-immunotherapy era (2001–2014) and 26 in the modern systemic therapy/immunotherapy era (2015–2025; Supplementary Table 6). Immunotherapy exposure was more common in the modern era (73.1% vs. 25.0%; *p* = 0.033). Median OS was 19.0 months in the earlier era and 26.0 months in the modern era, without a statistically significant difference by log-rank testing (*p* = 0.842).

### Overall survival (OS)

**Fig. 1:**
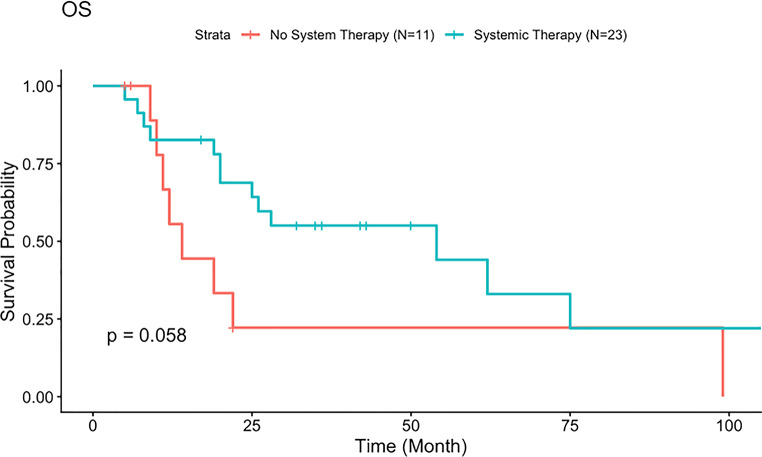
Overall survival by any systemic therapy exposure. Kaplan–Meier overall survival (OS) stratified by receipt of any systemic therapy (Tyrosine Kinase Inhibitor (TKI) and/or immunotherapy) versus none. Time origin is first GKRS, tick marks denote censoring. Groups were compared using the log-rank test

There were 22 deaths at the time of analysis. In Kaplan–Meier analysis, OS showed a trend toward difference by receipt of any systemic therapy (TKI and/or IO) versus none (log-rank *p* = 0.058) (Fig. 1). OS did not differ significantly by TKI exposure (log-rank *p* = 0.181) or immunotherapy exposure (log-rank *p* = 0.072) (Supplementary Fig. 1; Table 2).

**Table 2:** Overall survival by systemic therapy exposure

Exposure	Log-rank *p*	Univariate HR	95% CI	Univariate *p*
Any systemic (TKI and/or IO) vs. none	0.058	0.427	0.171–1.062	0.067
TKI exposure (yes vs. no)	0.181	0.555	0.230–1.338	0.190
Immunotherapy exposure (yes vs. no)	0.072	0.455	0.188–1.100	0.080

Covariate (adjusted model)		Adjusted HR	95% CI	Adjusted p
TKI		0.191	0.062–0.593	0.004
Age		1.054	1.004–1.107	0.034
KPS, per 10-point increase		0.37	0.21–0.63	< 0.001
Disease Burden		1.940	0.505–7.454	0.335

Kaplan–Meier log-rank comparisons and Cox proportional hazards models for overall survival (time from first GKRS to death). Exposures are time-agnostic (ever-exposed) systemic therapy variables (any systemic therapy, Tyrosine Kinase Inhibitor (TKI), Immunotherapy(IO)). The primary adjusted model is parsimonious (TKI, age, Karnofsky Performance Score (KPS), high disease burden) due to limited events

In univariate Cox regression, any systemic therapy was associated with lower hazard of death but did not meet conventional statistical significance (HR 0.427; 95% CI 0.171–1.062; *p* = 0.067) (Table 2). Similarly, immunotherapy exposure showed a directionally favorable but non-significant association (HR 0.455; 95% CI 0.188–1.100; *p* = 0.080) and TKI exposure was not significant in univariate analysis (HR 0.555; 95% CI 0.230–1.338; *p* = 0.190) (Table 2).

Given sample size and event counts, a parsimonious multivariable Cox model (TKI, age, KPS, disease burden) was used as the primary adjusted analysis. In this model, TKI exposure was associated with improved OS in the parsimonious adjusted model (adjusted HR 0.191; 95% CI 0.062–0.593; *p* = 0.004). Age was associated with worse OS (HR 1.054 per year; 95% CI 1.004–1.107; *p* = 0.034), whereas higher KPS was associated with improved OS (HR 0.37 per 10-point increase; 95% CI 0.21–0.63; *p* < 0.001). Disease burden was not significantly associated with OS (HR 1.940; 95% CI 0.505–7.454; *p* = 0.335) (Table 2).

### Patient-level intracranial treatment failure

There were 10 intracranial treatment failure events (patient-level). Time to intracranial treatment failure did not differ by TKI exposure (log-rank *p* = 0.22, Supplementary Fig. 2), immunotherapy exposure (*p* = 0.800, Supplementary Fig. 4), or any systemic therapy versus none (log-rank *p* = 0.692, Supplementary Fig. 5; Table 3).

**Table 3:** Patient-level intracranial treatment failure by systemic therapy exposure

Exposure	Log-rank *p*	Univariate HR	95% CI	Univariate *p*
Any systemic (TKI and/or IO) vs. none	0.692	0.753	0.182–3.109	0.695
TKI exposure (yes vs. no)	0.22	0.367	0.098–1.375	0.137
Immunotherapy exposure (yes vs. no)	0.800	1.193	0.301–4.728	0.802

Covariate (adjusted model)		Adjusted HR	95% CI	Adjusted p
TKI		0.481	0.107–2.152	0.338
Age		0.969	0.898–1.045	0.410
KPS		1.005	0.943–1.070	0.886
Disease burden		0.467	0.114–1.923	0.292

Kaplan–Meier log-rank comparisons and Cox models for patient-level intracranial treatment failure (time from first GKRS to intracranial failure). Exposures are time-agnostic systemic therapy variables. Adjusted model includes TKI, age, Karnofsky Performance Score (KPS), high disease burden. IO - Immunotherapy

In univariate Cox regression for intracranial treatment failure, associations were not statistically significant for any systemic therapy (HR 0.753; 95% CI 0.182–3.109; *p* = 0.695), TKI exposure (HR 0.367; 95% CI 0.098–1.375; *p* = 0.137), or immunotherapy exposure (HR 1.193; 95% CI 0.301–4.728; *p* = 0.802) (Table 3). In the parsimonious adjusted model (TKI, age, KPS, disease burden), TKI exposure was not associated with intracranial failure (adjusted HR 0.481; 95% CI 0.107–2.152; *p* = 0.338) (Table 3).

### Lesion-level local failure and cavity status

At the lesion level, there were 15 treatment failures among 87 lesions. Failure rates were substantially higher for cavity lesions (5/7 [71.4%]) compared with intact lesions (10/80 [12.5%]) (Fisher’s exact *p* = 0.001) (Table 4). Cavity lesions had substantially larger baseline volumes than intact lesions (median 9.71 [IQR 4.683–14.582] vs. 0.073 [IQR 0.024–0.412], p < 0.01) and higher Dmax (median 36.0 [IQR 33.35–38.8] vs. 24.4 [IQR 21.78–33.75], *p* = 0.012) (Table 4).

**Table 4:** Lesion-level treatment failure, cavity status, and early volumetric trajectory

Panel A. Descriptive lesion-level outcomes by cavity status				
Variable	Overall	Intact	Cavity	*p*-value
Lesions, n	87	80	7	—
Treatment failure, n (%)	15 (17.2%)	10 (12.5%)	5 (71.4%)	0.001
Initial GKRS volume, median (IQR)	0.1 (0.0–0.8)	0.1 (0.0–0.4)	9.7 (4.7–14.6)	< 0.001
Dmax, median (IQR)	25.0 (21.9–36.0)	24.4 (21.8–33.8)	36.0 (33.4–38.8)	0.012

Panel B. Prespecified clustered baseline/dosimetric GEE model for treatment failure (*N* = 87 lesions)				
Predictor	OR	95% CI	p-value	
Cavity, yes vs. no	9.92	1.44–68.27	0.020	
Log baseline volume	1.01	0.83–1.23	0.924	
Dmax, per Gy	1.05	0.99–1.12	0.096	

Panel C. Exploratory early volumetric trajectory model for subsequent treatment failure (*N* = 70 lesions with 6-month volume)				
Predictor	OR	95% CI	p-value	
6-month log volume ratio (ε = 0.01)	2.84	1.16–6.96	0.023	

Lesion-level outcomes comparing intact lesions versus postoperative cavities are shown in Panel A. Treatment failure is reported as n (%). Baseline volume and Dmax are reported as median (IQR). P-values reflect Fisher’s exact test for categorical variables and Wilcoxon rank-sum tests for continuous variables. Panel B shows generalized estimating equation (GEE) logistic regression evaluating predictors of lesion-level treatment failure with robust sandwich standard errors clustered by patient ID. Panel C shows the exploratory association between early volumetric trajectory and subsequent lesion-level treatment failure among lesions with available 6-month volumetric follow-up. Early volumetric trajectory was defined as the 6-month epsilon-stabilized log volume ratio ln{(V6m + ε)/(VGKRS + ε)}, ε = 0.01. Higher values indicate less regression or interval growth. GKRS, Gamma Knife radiosurgery; OR, odds ratio; CI, confidence interval; Dmax, maximum dose

**Fig. 2:**
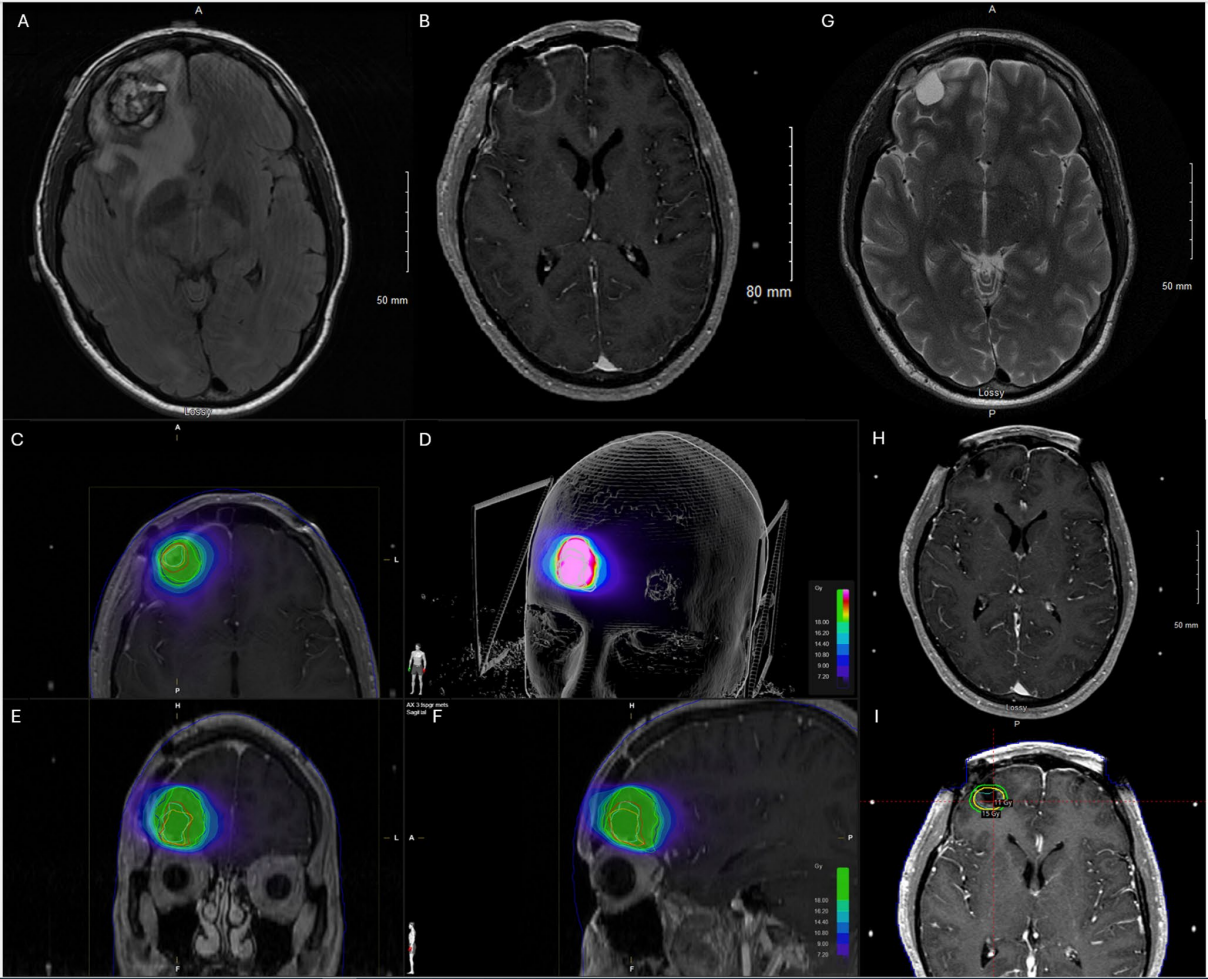
Illustrative postoperative cavity recurrence after Gamma Knife radiosurgery for renal cell carcinoma brain metastasis. **A**, Preoperative MRI demonstrating a right frontal renal cell carcinoma brain metastasis. **B**, Postoperative contrast-enhanced MRI obtained at the time of Gamma Knife radiosurgery (GKRS) planning, demonstrating the resection cavity. **C**–**F**, GKRS treatment planning images showing isodose distribution/dose cloud coverage of the postoperative cavity in axial (**C**), 3-dimensional (**D**), coronal (**E**), and sagittal views (**F**). **G**, Four-month post-GKRS MRI demonstrating no evidence of local recurrence. **H**, Eight-month post-GKRS MRI demonstrating a new nodular enhancing focus along the cavity margin, consistent with local recurrence. **I**, Salvage GKRS treatment plan for the recurrent nodular focus

An illustrative postoperative cavity case demonstrating initial GKRS dose distribution, interval local control, subsequent nodular cavity-margin recurrence, and salvage GKRS is shown in Fig. 2. To account for multiple lesions per patient, clustered lesion-level regression was performed using GEE logistic regression with robust sandwich standard errors clustered by patient ID (exchangeable working correlation; predictors: cavity status, log baseline volume, and Dmax). In this adjusted model, cavity status remained independently associated with treatment failure (OR 9.92; 95% CI 1.44–68.27; *p* = 0.020) (Table 4).

### Volumetric response and baseline correlates

Volumetric follow-up was available for 70 lesions at ~ 6 months, 36 at ~ 1 year, 17 at ~ 2 years, and 77 at last follow-up. Early volumetric trajectory was quantified as the 6-month epsilon-stabilized log volume ratio, with more negative values indicating greater lesion regression and higher values indicating less regression or interval growth. Among lesions with 6-month volumetric data (*n* = 70), 6-month log volume ratio was not significantly associated with Dmax (Spearman ρ = −0.075; *p* = 0.535) (Supplementary Fig. 3; Supplementary Table 2) or with baseline lesion volume (Spearman ρ = −0.177; *p* = 0.142) (Supplementary Table 2). Six-month log volume ratio also did not differ significantly by cavity status (*p* = 0.445), TKI exposure (*p* = 0.557), or immunotherapy exposure (*p* = 0.541) (Supplementary Table 2). In a sensitivity analysis restricted to one index lesion per patient (largest baseline lesion among those with 6-month data; *n* = 30), the association between Dmax and 6-month log volume ratio remained non-significant (Spearman ρ = −0.157; *p* = 0.408) (Supplementary Table 2).

### Early volumetric response and subsequent local failure

Among lesions with available 6-month volumetric follow-up (*n* = 70), early volumetric trajectory differed by subsequent lesion treatment failure. Lesions that ultimately failed demonstrated less early shrinkage at ~ 6 months (median epsilon-stabilized log volume ratio − 0.288 vs. − 0.669 for lesions without failure; *p* = 0.0023, Mann–Whitney U test) (Table 4). Among the 10 intact lesions that failed, baseline volume was median 0.63 cc (IQR 15.1), compared with median 0.07 cc (IQR 0.62) among intact lesions without failure. However, this difference did not reach statistical significance (Mann-Whitney U test, *p* = 0.06).

In a lesion-level patient-clustered GEE logistic regression model, higher (worse) 6-month log volume ratio was associated with increased odds of treatment failure (OR 2.84 per + 1 unit increase; 95% CI 1.16–6.96; *p* = 0.023) (Table 4). This association persisted in an index-lesion sensitivity analysis (largest baseline lesion per patient; *n* = 30), in which lesions that failed also demonstrated less early shrinkage (median − 0.347 vs. − 0.919; *p* = 0.0068) (Table 4).

## Discussion

This retrospective series describes GKRS outcomes for RCC brain metastases across the targeted-therapy and immune-checkpoint eras, with lesion-level volumetrics layered onto standard survival and intracranial endpoints. Three observations frame the interpretation - first, patient survival in this cohort appears dominated by systemic context and functional reserve: TKI exposure was associated with improved OS after parsimonious adjustment, alongside expected associations between older age, lower performance status, and worse survival. Second, intracranial behavior was not uniform within RCC brain metastases. Intact metastases demonstrated comparatively durable control, whereas postoperative cavities appeared to represent a higher-risk substrate, with markedly higher local failure, persisting after accounting for clustering and key lesion-level covariates - but the small number of cavity targets limits definitive inference. Third, while a single dosimetric scalar (Dmax) did not explain early volumetric response, the 6-month volumetric trajectory itself carried signal, lesions with poor early involution were more likely to ultimately fail locally, suggesting a practical imaging biomarker for earlier risk stratification.

RCC brain metastases occupy an awkward niche and remain clinically distinctive. The historical label of “radioresistant” largely reflects conventional fractionation experience [25–27], yet stereotactic approaches have consistently produced high rates of local control in RCC across multiple series, supporting SRS/GKRS as a backbone of intracranial management in this histology [10, 25, 28, 29]. The challenge is not whether RCC lesions can be controlled, but which lesions behave differently and why. RCC’s propensity for hemorrhage and complex post-treatment imaging evolution complicates response assessment and can blur the boundary between treatment effect, hemorrhagic change, and true progression, issues that become particularly relevant when interpreting cavity targets and volumetric endpoints [24, 30]. 

Prior retrospective series have established SRS/GKRS as an effective local therapy for RCC brain metastases, with generally favorable treated-lesion control despite the historical radioresistance of RCC. Earlier studies primarily defined the role of radiosurgery in local management [16, 25, 28, 29], whereas later series incorporated targeted therapy and, more recently, immune checkpoint inhibitor exposure [10, 17, 18]. The present study adds to this literature by combining systemic therapy-era patient-level outcomes with clustered lesion-level modeling, explicit evaluation of postoperative cavity targets, and MRI-based early volumetric trajectory after GKRS. (Table 5)

**Table 5:** Selected prior studies of stereotactic radiosurgery for renal cell carcinoma brain metastases and contribution of the current study

Study	Cohort / treatment context	Primary focus	Key contribution	Limitations / remaining gaps	Contribution of current study
Sheehan et al., 2003 [28]	RCC brain metastases treated with radiosurgery	Historical Gamma Knife outcomes	Established radiosurgery as an effective local therapy for RCC brain metastases, reporting high local control and clinically meaningful survival despite RCC radioresistance	Predated modern TKI and immunotherapy eras; limited contemporary systemic therapy context	Provides modern single-institution GKRS experience across targeted therapy and immunotherapy eras
Cochran et al., 2012 [16]	RCC brain metastases treated with Gamma Knife surgery, with/without targeted agents	Effect of targeted agents on outcomes after GKS	Suggested that targeted agents may improve local control and overall survival after GKS	Treatment timing and selection bias remain major limitations; limited immunotherapy-era relevance	Evaluates systemic therapy exposure in a later cohort including TKI and immunotherapy exposure
Lin et al., 2013 [29]	RCC and melanoma brain metastases treated with Gamma Knife SRS	Histology-specific dose-response comparison	Examined RCC radiosurgical dose-response and supported SRS efficacy in radioresistant histologies	Mixed-histology comparison; limited postoperative cavity and volumetric response assessment	Adds lesion-level RCC-specific modeling, including cavity status, Dmax, and early volumetric trajectory
Ippen et al., 2015 [25]	RCC brain metastases treated with SRS, surgery, and/or WBRT	Prognostic factors and treatment strategy	Reinforced the role of SRS as part of multimodality management for RCC brain metastases	Heterogeneous local therapies; limited modern systemic therapy integration	Focuses specifically on GKRS-treated lesions with patient-level and lesion-level outcomes
Wardak et al., 2019 [10]	38 patients with metastatic RCC and 243 brain metastases treated with SRS	SRS for multiple RCC brain metastases	Demonstrated high treated-lesion local control at 1 and 2 years and supported SRS even for multiple metastases	Less emphasis on postoperative cavities, clustered lesion-level predictors, or volumetric response	Adds clustered lesion-level analysis and evaluates postoperative cavity status as a distinct radiosurgical substrate
Juloori et al., 2020 [17]	RCC brain metastases treated with radiation and targeted therapies	Survival and intracranial outcomes with targeted therapy	Reported improved overall survival associated with targeted therapy in patients receiving intracranial radiation/SRS	Retrospective design with potential treatment-selection and era confounding	Confirms the clinical relevance of systemic therapy exposure while emphasizing limitations of time-agnostic exposure capture
Yomo et al., 2023 [18]	RCC brain metastases treated with SRS with/without immune checkpoint inhibitors	Immunotherapy combined with SRS	Addressed immunotherapy-era management and suggested benefit of immune checkpoint inhibitor integration with SRS	Retrospective propensity-weighted design; limited generalizability across institutions	Includes both TKI and immunotherapy exposure groups in a real-world GKRS cohort spanning systemic therapy eras
Current study	34 patients / 87 RCC brain metastases treated with GKRS from 2001–2025	Survival, intracranial treatment failure, lesion-level failure, cavity status, and MRI volumetric response	Integrates systemic therapy-era outcomes with clustered lesion-level modeling and early MRI volumetric trajectory; identifies postoperative cavities and poorer 6-month volumetric trajectory as clinically relevant signals	Retrospective single-institution design; small number of cavity targets; time-agnostic systemic therapy exposure; limited dose-volume metrics	Adds lesion-level cavity analysis and early volumetric trajectory assessment to the existing RCC SRS/GKRS literature

The systemic therapy findings should be interpreted with RCC-specific nuance. The adjusted association between TKI exposure and OS is biologically plausible in metastatic RCC, where extracranial disease trajectory typically drives survival; however, because systemic exposure was captured in a time-agnostic “ever exposed” fashion, causality cannot be inferred, and confounding by selection and era effects is likely. The agent distribution, dominated by nivolumab and cabozantinib, situates the cohort in contemporary RCC practice [31, 32]. Notably, cabozantinib has shown intracranial activity in RCC brain metastases cohorts [31], and IO-based regimens have demonstrated activity and feasibility in dedicated RCC brain metastasis subsets, supporting the relevance of these agents to the population studied.

Notably, we did not observe significant differences in patient-level intracranial treatment failure by systemic exposure. That negative result is still informative: it argues against over-interpreting the OS association as necessarily mediated by intracranial control. Instead, it supports a clinically familiar model in RCC - systemic therapy influences survival primarily via extracranial disease control, while radiosurgery provides durable lesion-level management for many patients. Although treatment era was associated with greater immunotherapy exposure, exploratory era-stratified analysis did not demonstrate a statistically significant OS difference; however, this comparison was limited by small sample size, differential follow-up, and overlap between era and systemic therapy availability.

The most clinically consequential intracranial signal in this study was the high failure rate in postoperative cavities compared with intact lesions. This finding aligns with the broader postoperative SRS literature emphasizing that cavities are dynamic, irregular targets with geometric uncertainty and potential microscopic extension along interfaces that are not well summarized by a single number such as Dmax [11–13, 33, 34]. 

Two interpretations are worth flagging for RCC specifically. First, RCC cavities may represent a biologically aggressive subset (symptomatic hemorrhagic or mass-effect lesions selected for surgery), so cavity status may proxy for more than geometry. Second, RCC’s propensity for hemorrhage and complex post-treatment imaging changes can blur the distinction between true recurrence, hemorrhagic evolution, and treatment effect - an issue that becomes magnified in irregular cavities [24, 30]. RCC cavities should be treated as high-surveillance targets where technique (margin strategy, coverage of dural contact, timing, and consideration of fractionation) may matter as much as prescription dose alone, more so in RCC. Data linking dural contact to recurrence risk after cavity SRS provide a mechanistic rationale for margin coverage considerations in select cases [35]. The illustrative cavity recurrence case in Fig. 2 highlights the practical challenge of interpreting and managing postoperative cavity targets, where nodular recurrence may emerge after an initially controlled postoperative bed.

The volumetric analyses add a useful layer to this risk-stratification theme. Dmax was not correlated with early volumetric change, which is unsurprising given that Dmax compresses heterogeneous dose distributions and does not capture coverage of critical interfaces, particularly relevant in cavities. More interestingly, early volumetric trajectory at ~ 6 months was associated with subsequent local failure in both clustered modeling and index-lesion sensitivity analysis, suggesting that the 6-month MRI may function as an early discriminator between lesions on a durable-control path and lesions that are quietly declaring resistance or under-coverage. This dovetails with the broader recognition that brain metastasis response assessment is nuanced and that standardized frameworks are needed to interpret imaging evolution in a clinically meaningful way [36–38]. 

In practice, the 6-month MRI may be an actionable fork: lesions that fail to demonstrate expected involution in RCC could be flagged for closer follow-up, multidisciplinary review, advanced imaging as needed, and earlier salvage consideration, rather than waiting for unequivocal progression.

This study is limited by its retrospective design, modest cohort size (especially for cavities), and the inability to model systemic therapy sequencing relative to GKRS, necessitating time-agnostic exposure definitions. Intracranial failure events at the patient level were few, constraining power and mandating parsimonious adjustment. Lesion-level analyses were appropriately clustered, but additional cavity-specific variables (e.g., dural contact, cavity dynamics, timing from surgery to GKRS) and richer dose–volume metrics would be important to incorporate in future work. The use of an approximately 6-month landmark MRI may underestimate the temporal precision of intracranial or lesion-level failure, particularly for failures occurring between imaging intervals. We examined Dmax as an exploratory scalar dosimetric variable because it was consistently available across the full historical GKRS registry. Established prognostic scores such as GPA could not be robustly reconstructed for all patients because several required variables were incompletely captured historically.

Volumetric interpretation of postoperative cavities is inherently complex. Postoperative cavities may contract over time independent of radiation effect and may persist as benign fluid-filled spaces after GKRS [38, 39]. In this retrospective analysis, cavity volumes were measured radiographically as treated targets, and persistent non-enhancing or benign-appearing fluid components could not be reliably separated from treatment-related or recurrent components across all historical scans. Therefore, volumetric response findings involving cavities should be considered cautiously.

## Conclusion

In RCC brain metastases treated with GKRS, we observed a separation between systemic survival determinants and intracranial lesion behavior. Postoperative cavities showed substantially higher local failure than intact lesions, even after clustered adjustment. While Dmax did not correlate with early volumetric change, early volumetric trajectory at ~ 6 months was associated with subsequent local failure, offering a pragmatic imaging signal for risk-adapted surveillance and salvage planning. These findings support treating RCC cavities as a distinct high-risk radiosurgical substrate and integrating early volumetric response into post-GKRS decision-making.

## Supplementary Information

Below is the link to the electronic supplementary material.

Supplementary Material 1

## Data Availability

The data that support the findings of this study are available from the corresponding author upon reasonable request.

## References

[1] Gray RE, Harris GT (2019) Renal Cell Carcinoma: Diagnosis and Management. Am Family Phys 99(3):179–184 30702258

[2] Rizzo A, Monteiro FSM, Mollica V et al (2024) Metastatic sites and clinical outcomes in renal cell carcinoma patients receiving immune-based combinations: the MOUSEION-08 study. Clin Exp Metastasis 42(1):9 39739072 10.1007/s10585-024-10327-w

[3] Motzer RJ, Agarwal N, Beard C et al (2011) Kidney cancer. J Natl Compr Canc Netw 9(9):960–977 21917622 10.6004/jnccn.2011.0082

[4] Ljungberg B, Hanbury DC, Kuczyk MA et al (2007) Renal cell carcinoma guideline. Eur Urol 51(6):1502–1510 17408850 10.1016/j.eururo.2007.03.035

[5] Gong J, Maia MC, Dizman N, Govindarajan A, Pal SK (2016) Metastasis in renal cell carcinoma: Biology and implications for therapy. Asian J Urol 3(4):286–292 29264197 10.1016/j.ajur.2016.08.006 PMC5730828

[6] Padala SA, Barsouk A, Thandra KC et al (2020) Epidemiology of Renal Cell Carcinoma. World J Oncol 11(3):79–87 32494314 10.14740/wjon1279 PMC7239575

[7] Gulati S, Barata PC, Elliott A et al (2024) Molecular analysis of primary and metastatic sites in patients with renal cell carcinoma. J Clin Invest 134(14):e176230 39007269 10.1172/JCI176230 PMC11245151

[8] Lwu S, Goetz P, Monsalves E et al (2013) Stereotactic radiosurgery for the treatment of melanoma and renal cell carcinoma brain metastases. Oncol Rep 29(2):407–412 23151681 10.3892/or.2012.2139 PMC3583599

[9] Hanson PW, Elaimy AL, Lamoreaux WT et al (2012) A concise review of the efficacy of stereotactic radiosurgery in the management of melanoma and renal cell carcinoma brain metastases. World J Surg Oncol 10(1):176 22931379 10.1186/1477-7819-10-176 PMC3502222

[10] Wardak Z, Christie A, Bowman A et al (2019) Stereotactic radiosurgery for multiple brain metastases from renal-cell carcinoma. Clin Genitourin Cancer 17(2):e273–e280 30595522 10.1016/j.clgc.2018.11.006 PMC6563340

[11] Alghamdi M, Hasan Y, Ruschin M et al (2018) Stereotactic radiosurgery for resected brain metastasis: Cavity dynamics and factors affecting its evolution. J Radiosurg SBRT 5(3):191–200 29988304 PMC6018046

[12] Atalar B, Choi CYH, Harsh R 4 et al (2013) Cavity volume dynamics after resection of brain metastases and timing of postresection cavity stereotactic radiosurgery. Neurosurgery 72(2):180–185 23149969 10.1227/NEU.0b013e31827b99f3

[13] Jarvis LA, Simmons NE, Bellerive M et al (2012) Tumor bed dynamics after surgical resection of brain metastases: implications for postoperative radiosurgery. Int J Radiat Oncol Biol Phys 84(4):943–948 22494581 10.1016/j.ijrobp.2012.01.067

[14] Semenescu LE, Kamel A, Ciubotaru V et al (2023) An Overview of Systemic Targeted Therapy in Renal Cell Carcinoma, with a Focus on Metastatic Renal Cell Carcinoma and Brain Metastases. Curr Issues Mol Biol 45(9):7680–7704 37754269 10.3390/cimb45090485 PMC10528141

[15] Johnson AG, Ruiz J, Hughes R et al (2015) Impact of systemic targeted agents on the clinical outcomes of patients with brain metastases. Oncotarget 6(22):18945–18955 26087184 10.18632/oncotarget.4153 PMC4662466

[16] Cochran DC, Chan MD, Aklilu M et al (2012) The effect of targeted agents on outcomes in patients with brain metastases from renal cell carcinoma treated with Gamma Knife surgery. J Neurosurg 116(5):978–983 22385005 10.3171/2012.2.JNS111353 PMC3791504

[17] Juloori A, Miller JA, Parsai S et al (2020) Overall survival and response to radiation and targeted therapies among patients with renal cell carcinoma brain metastases. J Neurosurg 132(1):188–196 30660120 10.3171/2018.8.JNS182100

[18] Yomo S, Oda K, Oguchi K (2023) Effectiveness of immune checkpoint inhibitors in combination with stereotactic radiosurgery for patients with brain metastases from renal cell carcinoma: inverse probability of treatment weighting using propensity scores. J Neurosurg 138(6):1591–1599 36308485 10.3171/2022.9.JNS221215

[19] Franzese C, Marvaso G, Francolini G et al (2021) The role of stereotactic body radiation therapy and its integration with systemic therapies in metastatic kidney cancer: a multicenter study on behalf of the AIRO (Italian Association of Radiotherapy and Clinical Oncology) genitourinary study group. Clin Exp Metastasis 38(6):527–537 34748125 10.1007/s10585-021-10131-w

[20] Takemura K, Lemelin A, Ernst MS et al (2024) Outcomes of Patients with Brain Metastases from Renal Cell Carcinoma Receiving First-line Therapies: Results from the International Metastatic Renal Cell Carcinoma Database Consortium. Eur Urol 86(6):488–492 38290965 10.1016/j.eururo.2024.01.006

[21] Vonderhaar-Meister EP, Lin TA, Li A et al (2026) Optimal timing of initial surveillance MRI following treatment of brain metastases with stereotactic radiation: A comparison of six weeks versus three months. Cureus 18(1):e102720 41777995 10.7759/cureus.102720 PMC12952540

[22] Gondi V, Bauman G, Bradfield L et al (2022) Radiation therapy for brain metastases: An ASTRO clinical practice guideline. Pract Radiat Oncol 12(4):265–282 35534352 10.1016/j.prro.2022.02.003

[23] Lin NU, Lee EQ, Aoyama H et al (2015) Response assessment criteria for brain metastases: proposal from the RANO group. Lancet Oncol 16(6):e270–e278 26065612 10.1016/S1470-2045(15)70057-4

[24] Ressler HW, Cramer CK, Isom S et al (2024) Brain metastases from renal cell carcinoma: Effects of novel systemic agents on brain metastasis outcomes. Clin Neurol Neurosurg 238(108191):108191 38422744 10.1016/j.clineuro.2024.108191

[25] Ippen FM, Mahadevan A, Wong ET, Uhlmann EJ, Sengupta S, Kasper EM (2015) Stereotactic Radiosurgery for renal cancer brain metastasis: Prognostic factors and the role of whole-brain radiation and surgical resection. J Oncol 2015:636918 26681942 10.1155/2015/636918 PMC4668321

[26] Blanco AI, Teh BS, Amato RJ (2011) Role of radiation therapy in the management of renal cell cancer. Cancers (Basel) 3(4):4010–4023 24213122 10.3390/cancers3044010 PMC3763407

[27] Haque W, Verma V, Butler EB, Teh BS (2018) Utilization of stereotactic radiosurgery for renal cell carcinoma brain metastases. Clin Genitourin Cancer 16(4):e935–e943 29680768 10.1016/j.clgc.2018.03.015

[28] Sheehan JP, Sun MH, Kondziolka D, Flickinger J, Lunsford LD (2003) Radiosurgery in patients with renal cell carcinoma metastasis to the brain: long-term outcomes and prognostic factors influencing survival and local tumor control. J Neurosurg 98(2):342–349 12593621 10.3171/jns.2003.98.2.0342

[29] Lin HY, Watanabe Y, Cho LC et al (2013) Gamma knife stereotactic radiosurgery for renal cell carcinoma and melanoma brain metastases-comparison of dose response. J Radiosurg SBRT 2(3):193–207 29296362 PMC5658811

[30] Internò V, De Santis P, Stucci LS et al (2021) Prognostic factors and current treatment strategies for renal cell carcinoma metastatic to the brain: An overview. Cancers (Basel) 13(9):2114 33925585 10.3390/cancers13092114 PMC8123796

[31] Hirsch L, Martinez Chanza N, Farah S et al (2021) Clinical activity and safety of cabozantinib for brain metastases in patients with renal cell carcinoma. JAMA Oncol 7(12):1815–1823 34673916 10.1001/jamaoncol.2021.4544 PMC8532040

[32] Emamekhoo H, Olsen MR, Carthon BC et al (2022) Safety and efficacy of nivolumab plus ipilimumab in patients with advanced renal cell carcinoma with brain metastases: CheckMate 920. Cancer 128(5):966–974 34784056 10.1002/cncr.34016 PMC9298991

[33] Marchan EM, Peterson J, Sio TT et al (2018) Postoperative cavity stereotactic radiosurgery for brain metastases. Front Oncol 8:342 30234013 10.3389/fonc.2018.00342 PMC6127288

[34] Redmond KJ, De Salles AAF, Fariselli L et al (2021) Stereotactic Radiosurgery for Postoperative Metastatic Surgical Cavities: A Critical Review and International Stereotactic Radiosurgery Society (ISRS) Practice Guidelines. Int J Radiat Oncol Biol Phys 111(1):68–80 33891979 10.1016/j.ijrobp.2021.04.016

[35] Susko M, Yu Y, Ma L et al (2019) Preoperative Dural contact and recurrence risk after surgical cavity stereotactic radiosurgery for brain metastases: New evidence in support of consensus guidelines. Adv Radiat Oncol 4(3):458–465 31360800 10.1016/j.adro.2019.03.002 PMC6639748

[36] Ramakrishnan D, von Reppert M, Krycia M et al (2023) Evolution and implementation of radiographic response criteria in neuro-oncology. Neurooncol Adv 5(1):vdad118 37860269 10.1093/noajnl/vdad118 PMC10584081

[37] Douri K, Iorio-Morin C, Mercure-Cyr R et al (2023) Response assessment in brain metastases managed by stereotactic radiosurgery: A reappraisal of the RANO-BM criteria. Curr Oncol 30(11):9382–9391 37999099 10.3390/curroncol30110679 PMC10670467

[38] Goulenko V, Sinha S, Gulzar B et al (2026) Longitudinal remodeling of brain metastasis resection cavities after adjuvant gamma knife radiosurgery. J Neurooncol 177(3). 10.1007/s11060-026-05567-7 10.1007/s11060-026-05567-7 PMC13124882 42047864

[39] Shah JK, Potts MB, Sneed PK, Aghi MK, McDermott MW (2016) Surgical cavity constriction and local progression between resection and adjuvant radiosurgery for brain metastases. Cureus 8(4):e575 27226936 10.7759/cureus.575 PMC4873317

