# Supplementary material for: Gamma knife radiosurgery for renal cell carcinoma brain metastases across systemic therapy eras: survival, intracranial failure, and lesion-level predictors
Source: J Neurooncol. 2026 Jul 3;178(3):87. doi: 10.1007/s11060-026-05692-3 (PMC13328143; doi:10.1007/s11060-026-05692-3)
Supplement: Supplementary file 1 — Supplementary Material 1 [file 11060_2026_5692_MOESM1_ESM.docx]

Supplementary Materials

| **Therapy class** | **Agent** | **Patients (n)** | **Patients (%)** |
| --- | --- | --- | --- |
| TKI / Targeted Agent | Cabozantinib | 11 | 32.4 |
| TKI / Targeted Agent | Sunitinib | 4 | 11.8 |
| TKI / Targeted Agent | Axitinib | 3 | 8.8 |
| TKI / Targeted Agent | Lenvatinib | 2 | 5.9 |
| TKI / Targeted Agent | Pazopanib | 1 | 2.9 |
| TKI / Targeted Agent | Sorafenib | 1 | 2.9 |
| TKI / Targeted Agent | Tivozanib | 1 | 2.9 |
| Other targeted / anti-VEGF | Bevacizumab | 1 | 2.9 |
| Immunotherapy (checkpoint inhibitor) | Nivolumab | 18 | 52.9 |
| Immunotherapy (checkpoint inhibitor) | Ipilimumab | 11 | 32.4 |
| Immunotherapy (checkpoint inhibitor) | Pembrolizumab | 4 | 11.8 |
| Immunotherapy (checkpoint inhibitor) | Atezolizumab | 1 | 2.9 |

Supplementary Table 1: Systemic therapy agents represented in the cohort (patient-level, ever-exposed classification). Systemic therapy agents recorded in the institutional registry among patients treated with GKRS for intracranial RCC metastases (N = 34). Counts reflect a patient-level “ever exposed” classification (a patient may be counted under multiple agents). Agents are grouped as TKI/targeted therapy, checkpoint inhibitor immunotherapy, and other targeted/anti-VEGF therapy. Bevacizumab is reported under “other targeted/anti-VEGF” rather than targeted therapy.

Supplementary Table 2. Baseline and treatment correlates of early volumetric response. Six-month volumetric response was defined as epsilon-stabilized log volume ratio ln{(𝑉_6𝑚_+𝜀)/(𝑉_GKRS_+𝜀)}, ε=0.01.Correlations were assessed using Spearman’s rank correlation (reported for all eligible lesions and an index-lesion sensitivity analysis). Group comparisons were performed using Wilcoxon rank-sum tests among lesions with available 6-month volume. Results are shown for all eligible lesions and for a sensitivity analysis restricted to one index lesion per patient (largest baseline lesion).

| **Predictor** | **Analysis set** | **N** | **Spearman ρ** | **p-value** |
| --- | --- | --- | --- | --- |
| Dmax vs log volume ratio | All lesions with 6m volume | 70 | −0.075 | 0.535 |
| Dmax vs log volume ratio | Index lesion per patient | 30 | −0.157 | 0.408 |
| Log baseline volume vs log volume ratio | All lesions with 6m volume | 70 | −0.177 | 0.142 |
| Log baseline volume vs log volume ratio | Index lesion per patient | 30 | 0.111 | 0.558 |
|  |  |  |  |  |
| **Grouping variable** | **Comparison** | | **N** | **p-value** |
| Cavity status | cavity vs intact | | 70 | 0.445 |
| TKI exposure | yes vs no | | 70 | 0.557 |
| Immunotherapy exposure | yes vs no | | 70 | 0.541 |
| Any systemic therapy | yes vs no | | 70 | 0.850 |

| **Variable** | **Earlier era / pre-immunotherapy era, 2001–2014 (N=8)** | **Modern systemic therapy / immunotherapy era, 2015–2025 (N=26)** | **p-value** |
| --- | --- | --- | --- |
| Patients, n | 8 | 26 | — |
| Treated lesions, n | 23 | 64 | — |
| Age, years, median (IQR) | 65.5 (61.5–69.3) | 58.0 (54.0–66.3) | 0.208 |
| Male sex, n (%) | 6 (75.0%) | 17 (65.4%) | 1.000 |
| KPS, median (IQR) | 80.0 (77.5–80.0) | 80.0 (70.0–90.0) | 0.832 |
| ECOG, median (IQR) | 1.0 (1.0–1.0) | 1.0 (0.0–1.0) | 0.565 |
| Surgery, n (%) | 1 (12.5%) | 6 (23.1%) | 1.000 |
| High disease burden, n (%) | 8 (100.0%) | 19 (73.1%) | 0.160 |
| Smoking history, n (%) | 4 (50.0%) | 13 (50.0%) | 1.000 |
| Any systemic therapy, n (%) | 4 (50.0%) | 19 (73.1%) | 0.388 |
| TKI exposure, n (%) | 4 (50.0%) | 14 (53.8%) | 1.000 |
| Immunotherapy exposure, n (%) | 2 (25.0%) | 19 (73.1%) | 0.033 |
| Deaths, n (%) | 8 (100.0%) | 14 (53.8%) | 0.030 |
| Median OS, months | 19.0 | 26.0 | 0.842 |

Supplementary Table 6. Treatment era was defined according to date of first GKRS as earlier era/pre-immunotherapy era, 2001–2014, versus modern systemic therapy/immunotherapy era, 2015–2025. Continuous variables are reported as median (IQR) and compared using Wilcoxon rank-sum tests. Categorical variables are reported as n (%) and compared using Fisher’s exact tests. Median OS was estimated using Kaplan–Meier analysis and compared using the log-rank test. This analysis was exploratory and was not incorporated into the primary adjusted model because of limited sample size and overlap between treatment era and systemic therapy availability.

Supplementary Figures -


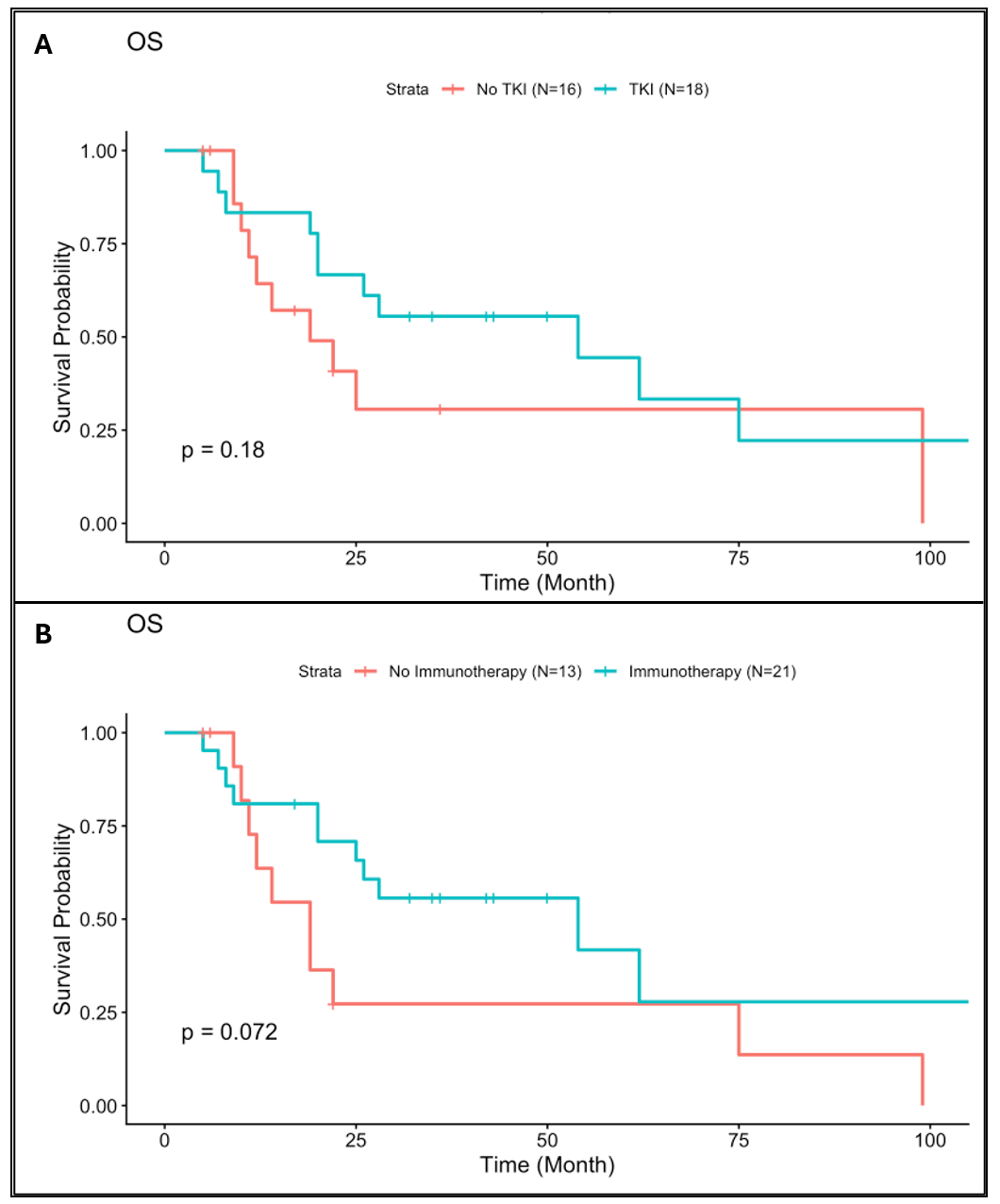


Supplementary Fig 1: Overall survival (OS) by systemic therapy class.(A) Overall survival by Tyrosine Kinase Inhibitor (TKI) exposure, (B) Overall survival by immunotherapy exposure. Kaplan–Meier OS stratified by (A) TKI exposure and (B) immunotherapy exposure (time-agnostic “ever exposed”). Time origin is first GKRS, tick marks denote censoring.


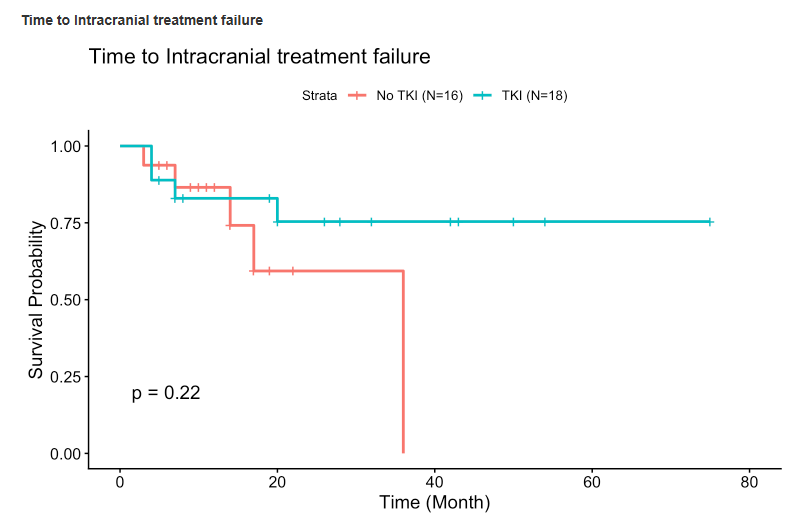


Supplementary Fig 2: Intracranial treatment failure-free survival by Tyrosine Kinase Inhibitor (TKI) exposure. Kaplan–Meier intracranial treatment failure-free survival stratified by TKI exposure. Time origin is first GKRS, and tick marks denote censoring. Groups were compared using the log-rank test.


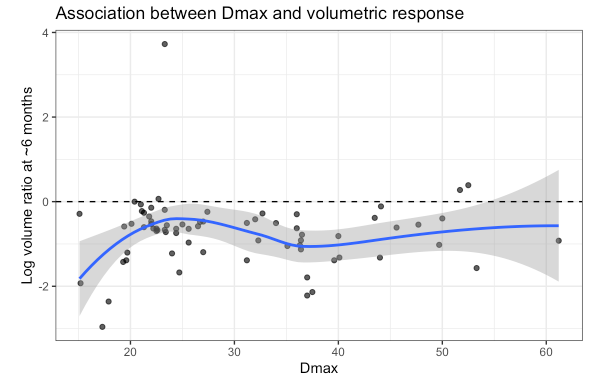


Supplementary Fig 3: Dmax and 6-month volumetric response.Scatter plot showing the association between Dmax and 6-month volumetric response among lesions with available 6-month follow-up. Volumetric response was calculated as an epsilon-stabilized log volume ratio: ln{(𝑉_6𝑚_+𝜀)/(𝑉_GKRS_+𝜀)}, ε=0.01. Association was assessed using Spearman’s rank correlation (Spearman ρ = −0.075; p = 0.535). Each point represents one treated lesion.


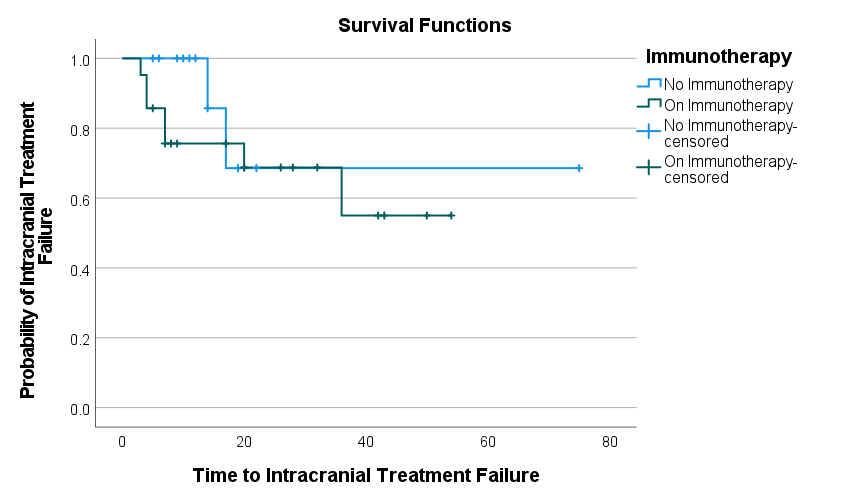


Supplementary Figure 4: Intracranial treatment failure-free survival by immunotherapy exposure. Kaplan–Meier intracranial treatment failure-free survival stratified by immunotherapy exposure (time-agnostic classification). Time origin is first GKRS, tick marks denote censoring. Groups compared using the log-rank test


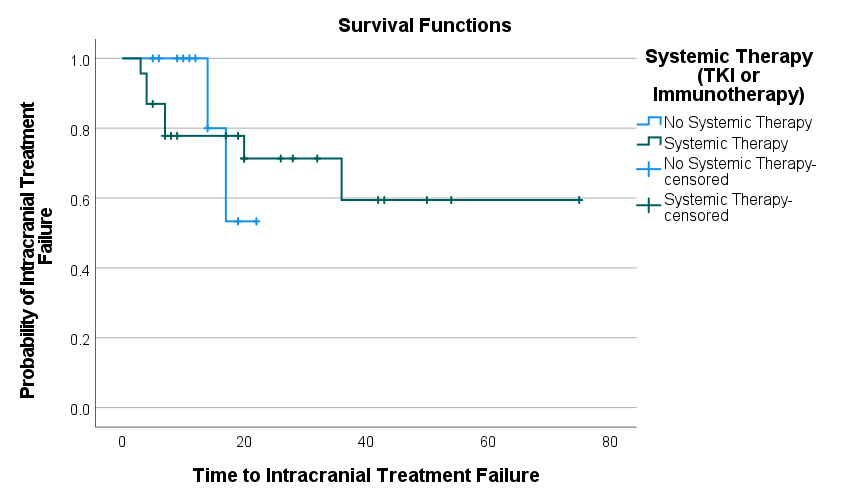


Supplementary Figure 5: Intracranial treatment failure-free survival by any systemic therapy exposure. Kaplan–Meier intracranial treatment failure-free survival stratified by receipt of any systemic therapy (Tyrosine Kinase Inhibitor (TKI) and/or immunotherapy) versus none (time-agnostic classification). Time origin is first GKRS, tick marks denote censoring. Groups compared using the log-rank test.
